# Guided Self‐Help Treatment for Children and Young People With Eating Disorders: A Proof‐Of‐Concept Pilot Study

**DOI:** 10.1002/erv.3171

**Published:** 2025-01-02

**Authors:** Emily Davey, Rachel Bryant‐Waugh, Sophie D. Bennett, Nadia Micali, Julian Baudinet, Anna Konstantellou, Sam Clark‐Stone, Amelia Green, Roz Shafran

**Affiliations:** ^1^ UCL Great Ormond Street Institute of Child Health University College London London UK; ^2^ Maudsley Centre for Child and Adolescent Eating Disorders South London and Maudsley NHS Foundation Trust London UK; ^3^ Department of Child and Adolescent Psychiatry Institute of Psychiatry, Psychology & Neuroscience King's College London London UK; ^4^ Department of Psychology Institute of Psychiatry, Psychology & Neuroscience King's College London London UK; ^5^ Center for Eating and Feeding Disorders Research Mental Health Center Ballerup Copenhagen University Hospital—Mental Health Services CPH Copenhagen Denmark; ^6^ Centre for Research in Eating and Weight Disorders (CREW) Institute of Psychiatry, Psychology & Neuroscience King's College London London UK; ^7^ The Eating Disorders Service Gloucestershire Health and Care NHS Foundation Trust Cheltenham UK; ^8^ Research and Development Gloucestershire Health and Care NHS Foundation Trust Cheltenham UK

**Keywords:** children and young people, eating disorders, guided self‐help, low intensity, pilot study

## Abstract

**Objective:**

To conduct a proof‐of‐concept pilot study of a CBT guided self‐help intervention for children and young people with eating disorders.

**Method:**

Children and young people were recruited from two outpatient eating disorder services in England. They received a CBT guided self‐help intervention consisting of eight modules and weekly support sessions. Clinical outcomes (eating disorder psychopathology and associated impairment, changes in %median BMI, depression, anxiety, and behavioural difficulties) were assessed at baseline and post‐intervention (12 weeks). Qualitative data were collected for future intervention refinement.

**Results:**

Six female adolescents (aged 13–17) received the CBT guided self‐help intervention. All participants completed a minimum of six modules and six support sessions. Quantitative and qualitative feedback suggested that the intervention was acceptable. From baseline to post‐intervention, there was a reduction in eating disorder psychopathology and impairment, along with an increase in %median BMI. Outcomes for depression, anxiety and behavioural difficulties were mixed.

**Conclusions:**

The CBT guided self‐help intervention was feasibly implemented, acceptable to participants, and showed potential to produce clinical benefits. While promising, these findings are preliminary and derived from a small, non‐randomised sample of White female adolescents. More rigorous evaluation with a randomised design and a larger, representative sample is warranted.

**Summary**

- This is the first study to evaluate a CBT guided self‐help intervention in a transdiagnostic sample of children and young people with eating disorders.
- The intervention was feasible to deliver, with all participants completing at least six modules and six support sessions. Quantitative and qualitative feedback suggested the intervention was acceptable.
- The intervention demonstrates potential for reducing eating disorder psychopathology and associated impairment.

## 1 Introduction

Eating disorders are serious mental health conditions associated with high levels of medical and psychosocial disability (Micali and Herle 2023). These conditions can significantly interfere with an individual's education, work, health‐related quality of life, psychological wellbeing, and interpersonal functioning (Beat 2015; Hay et al. 2017; Streatfeild et al. 2021). Eating disorders typically begin during adolescence (Solmi et al. 2022), and are prevalent among those who present to child and adolescent mental health services in the UK (National Collaborating Centre for Mental Health 2015). The COVID‐19 pandemic has had profound effects on young people with eating disorders, with worsened symptom severity and an increased incidence of diagnoses (Katzman 2021). Recent findings from the Mental Health of Children and Young people (MHCYP) survey in England estimate that 2.6% of young people aged 11%–16%, and 12.5% of those aged 17–19, have a diagnosable eating disorder (Newlove‐Delgado et al. 2023).

Effective psychological treatments for children and young people with eating disorders exist (Datta et al. 2023), such as family based treatment (FBT; Lock and Le Grange 2012) and enhanced cognitive behavioural therapy (CBT‐E; Dalle Grave and Calugi 2020). However, these interventions are resource‐intensive and services cannot keep up with the unprecedented demand (NHS England 2024). In England, the National Health Service (NHS) set a standard requiring that 95% of children and young people referred for assessment or treatment for an eating disorder should begin receiving treatment within 1 week for urgent cases and within 4 weeks for routine or non‐urgent cases (NHS England 2015). However, the most recent data from October 2023 to December 2023 indicates that only 64% of urgent cases and 79% of routine cases met these standards, falling short of the 95% target (NHS England 2024).

This unmet need for treatment among children and young people with eating disorders is concerning given that a long duration of untreated eating disorder can lead to a protracted illness course and poorer health outcomes (Austin et al. 2021; Pehlivan et al. 2022). Ultimately, there is a shortage of mental health professionals available to provide specialist eating disorder treatment to the number of children and young people that need it (Kazdin 2023). It is crucial that psychological treatment evolves away from time‐ and cost‐intensive therapist‐led formats to ensure that treatment is accessible within resource constraints, whilst maintaining their effectiveness (Kazdin, Fitzsimmons‐Craft, and Wilfley 2017).

Guided self‐help interventions are a promising option to increase access to psychological treatment. In the UK, CBT guided self‐help is recommended as the first‐line treatment for adults with bulimia nervosa and binge eating disorder (National Institute for Health and Care Excellence 2017). Additionally, CBT guided self‐help has proven efficacy for treating anxiety disorders in children and young people, regardless of the specific diagnosis (Bennett et al. 2019). Given the high comorbidity between eating disorders and anxiety (Hambleton et al. 2022), it is reasonable to hypothesise that these interventions could also benefit young people with eating disorders.

Although research in this area remains limited, existing evidence supports the effectiveness of guided self‐help for young people with eating disorders. For example, Schmidt et al. (2007) found that CBT guided self‐care led to a more rapid reduction in bingeing and was more cost‐effective than family therapy for adolescents with bulimia nervosa. Emerging evidence also suggests that guided self‐help based on FBT principles (GSH‐FBT) may be effective for adolescents with anorexia nervosa. Both GSH‐FBT (Lock et al. 2021) and its multi‐family variant (MF‐GSH‐FBT; Matheson et al. 2024) have been shown to produce clinical improvements, including weight gain and reductions in eating‐related cognitions, as well as anxiety and depression symptoms (Lock et al. 2021; Matheson et al. 2024). However, these existing guided self‐help interventions focus on one disorder and thereby forego the advantages of a transdiagnostic approach.

Transdiagnostic interventions offer significant benefits by addressing the heterogeneity and comorbidity often seen in real‐world settings (Levinson et al. 2022; Schaeuffele et al. 2021). This approach has shown effectiveness in adults with eating disorders (e.g., Atwood and Friedman 2020; C. G. Fairburn et al. 2003; Fitzsimmons‐Craft et al. 2020; Vollert et al. 2024). Building on this and the demonstrated efficacy of CBT guided self‐help in adults with eating disorders and for young people with anxiety disorders, we developed a transdiagnostic CBT guided self‐help intervention for children and young people with eating disorders. This intervention was developed using a common elements approach across the three pillars of evidence‐based practice (Davey et al. 2024a).

The aim of this study was to conduct a proof‐of‐concept pilot study to evaluate the feasibility, acceptability and preliminary effectiveness of the intervention in a sample of children and young people with a range of eating disorders. It was hypothesised that the intervention would be feasible and acceptable to young people and parents, and that young people would show improvements in symptomatology and impairment.

## 2 Methods

This study received ethical approval from the West of Scotland Research Ethics Committee 5 (approval number: 23/WS/0097). The study protocol has been published (Davey et al. 2024b) and registered with the ISRCTN registry (ISRCTN16038125). The reporting of this study aligns with the CONSORT extension checklist for pilot and feasibility trials (Eldridge et al. 2016). The full methodology is outlined in Davey et al. (2024b).

### 2.1 Study Design

A single‐arm, proof‐of‐concept pilot study was conducted. Proof‐of‐concept pilot studies focus on clinical benefits rather than statistical effectiveness (Czajkowski et al. 2015). As formal sample size calculations are unnecessary for proof‐of‐concept studies (Czajkowski et al. 2015), the number of children and young people recruited to receive the CBT guided self‐help intervention was not pre‐specified.

### 2.2 Participants

Participants were included if they lived in the UK, were between the ages of 11 and 19, and had a threshold eating disorder (anorexia nervosa, bulimia nervosa, binge eating disorder, other specified feeding or eating disorder [OSFED]) or subthreshold eating disorder. Participants were excluded from the study if they were at acute risk (e.g., ongoing rapid weight loss, very low mood, high medical or psychiatric risk, acute suicidality, recurrent or potentially life limiting self‐harm, or significant safeguarding concerns), were currently receiving overlapping psychological treatment, had a recent change in psychotropic medication dosage within the preceding 2 months, or were unable to access the intervention due to insufficient English proficiency, intellectual disability, or lack of access to a laptop or smartphone.

### 2.3 Procedure

Participants were recruited via two specialist eating disorder services in England: The Maudsley Centre for Child and Adolescent Eating Disorders (Site 1) and The Gloucestershire Eating Disorders Service (Site 2). For safety reasons, clinicians at each service exercised their professional judgement to assess the suitability of children and young people for a CBT guided self‐help intervention. At Site 1, this judgement was applied broadly to all children and young people with threshold eating disorders; referrals were limited to young people who had disordered eating but did not meet the diagnostic criteria for an eating disorder and were being discharged from the service. Site 2 offered the intervention to routine cases on the waiting list for FBT.

Young people and parents interested in the study provided consent/assent to participate and completed a short screening questionnaire to determine their eligibility. Once eligibility was confirmed, participants completed a baseline assessment before starting the intervention.

All participants received the Short Psychological Intervention for Children and adolescents with Eating disorders (SPICE) programme, a transdiagnostic, guided self‐help intervention based on CBT principles (see Davey et al. (2024a) for process of intervention development). The treatment manual consists of eight modules and covers the core components of CBT for eating disorders, including psychoeducation, reducing eating disorder behaviours, improving body image, addressing shape checking and avoidance, challenging negative thoughts, regulating emotions and preventing relapse (C. G. Fairburn 2008; Waller et al. 2019). It also covers low self‐esteem and the impact of social media in body image concerns (Choukas‐Bradley et al. 2022). See Table 1 for an overview of each module. The intervention was delivered through an interactive Portable Document Format (PDF) workbook. Each module had its own PDF workbook and accompanying home practice tasks.

**TABLE 1:** CBT guided self‐help for children and young people with eating disorders (SPICE) treatment modules.

Module		Description
1.	Understanding my eating difficulties	The aim of this module is to provide key information about eating disorders, including maintaining factors, and to help the young person think about what is keeping their own eating difficulties going. The young person also identifies goals for the intervention.
2.	Eating more regularly	The aim of this module is to help the young person understand the relationship between what they are eating and their energy levels. It also introduces ways to improve the structure of their eating and the types of food that they eat.
3.	Reducing dieting	The aim of this module is to help the young person identify and challenge any strict diet rules that are maintaining their eating difficulties, including rules around when to eat, what to eat and how much to eat.
4.	Doing things differently	The aim of this module is to provide the young person with some strategies to reduce and manage weight control behaviours, such as self‐induced vomiting, laxatives and exercising excessively.
5.	Body image and social media	The aim of this module is to provide some strategies to help the young person tackle concerns around their body image. It also discusses the role of social media in body image and ways to use social media in a more positive way.
6.	Learning to feel good about myself	The aim of this module is to provide the young person with some effective ways to improve their self‐esteem.
7.	Managing emotional triggers	The aim of this module is to explain the link between events, emotions and eating. It helps the young person to consider healthier ways to cope, including how to solve day‐to‐day problems.
8.	Planning for the future	The aim of this module is to help the young person maintain the progress they have made. It supports the young person to develop a plan for managing slips or setbacks which may happen in the future.

Participants were offered weekly support sessions via telephone or video call with a guide, each scheduled to last approximately 30 min. Participants were asked to read the relevant module and complete between‐session tasks prior to each support session. While each module was designed to have one accompanying support session, the number of sessions per module was adjusted according to treatment priorities. For example, if a participant required additional time to practice skills, a top‐up was offered the following week. The young person's parent/carer was encouraged, but not required, to attend these support sessions. All parents received a copy of the intervention materials to help them understand the content and to support their child's progress, regardless of their attendance in sessions.

Each support session was delivered by the first author (ED), a paraprofessional who was doing her doctoral research in child and adolescent eating disorders. ED received weekly supervision from her supervisor (RS) throughout the research and intervention process to ensure adherence to the agreed protocol. RS is a Consultant Clinical Psychologist with considerable experience in CBT for eating disorders. The guide took on a facilitative role during the support sessions. The aim of their guidance was to enhance motivation, troubleshoot problems that arise and refer participants to the intervention content to enhance knowledge and skills usage.

In the event of no progress, deterioration in wellbeing, or the emergence of risk and/or safeguarding concerns, the participant's GP and other relevant professionals involved in their care (e.g., the referring Eating Disorders Service) were contacted as deemed necessary. Participants were able to withdraw from the study at any time.

Post‐intervention measures were collected 12 weeks after the baseline assessment to account for any delays in treatment completion, such as session rescheduling, and to ensure a standardised data collection timepoint. A qualitative interview was also conducted at this 12‐week interval. The entire study was conducted remotely, and all measures were completed online. This remote approach was chosen to increase accessibility for participants.

### 2.4 Measures

#### 2.4.1 Feasibility and Acceptability

The feasibility of the intervention and study procedures were evaluated by examining referral and uptake rates, session attendance, intervention completion/attrition, and measure completion. Participants and their parents completed a seven‐item acceptability questionnaire adapted from Creswell et al. (2010) at the post‐intervention assessment.

#### 2.4.2 Session‐By‐Session Measures

Participants completed weekly questionnaires throughout the intervention, including Goal Based Outcomes (GBOs; Law and Jacob 2015), the Eating Disorder‐15 for Youth (ED‐15‐Y; Accurso and Waller 2021), and the Strengths and Difficulties Questionnaire Session by Session (SDQ SxS; Hall et al. 2015).

In Module One of the intervention, participants identified three intervention goals relating to their eating difficulties which were subsequently refined in collaboration with the guide during the first support session. Progress was rated weekly on a scale from 0 (indicating no progress) to 10 (indicating goal achieved). GBOs (Law and Jacob 2015) have been shown to improve treatment retention, clinical outcomes, and client progress (Delgadillo et al. 2018; Tryon, Birch, and Verkuilen 2018). The ED‐15‐Y (Accurso and Waller 2021) was used to assess weekly changes in eating attitudes and behaviours throughout the intervention, and the SDQ SxS (Hall et al. 2015), a non‐symptom‐specific measure, was also employed to evaluate participant progress.

#### 2.4.3 Baseline and Post‐Intervention Measures

Eating disorder psychopathology was assessed at baseline and post‐intervention using the Eating Disorder Examination Questionnaire (EDE‐Q; C. Fairburn and Beglin 2008), Clinical Impairment Assessment (CIA; Bohn et al. 2008), and % median Body Mass Index (%mBMI; calculated from relevant EDE‐Q data). The EDE‐Q global score was the primary outcome. Depression and anxiety symptomology were measured at the same timepoints using the Revised Child Anxiety and Depression Scale (RCADS; Chorpita et al. 2000) and the Strengths and Difficulties Questionnaire (SDQ; Goodman 1997). Parents completed the parent‐report version of the EDE‐Q (PEDE‐Q; Loeb 2008), the RCADS (Chorpita et al. 2000) and the SDQ (Goodman 1997). Information on the psychometric properties of each measure can be found in Davey et al. (2024b).

#### 2.4.4 Qualitative Feedback

Participants and their parents were invited to take part in a one‐to‐one qualitative interview with an independent research assistant. The interviews were guided by a topic guide (Supporting Information S1) and covered what families found helpful or unhelpful about the intervention, their views on the mode, content and structure of the treatment, and their suggestions for improvement.

### 2.5 Analyses

All quantitative analyses were conducted using IBM SPSS Statistics 29. Descriptive statistics were employed to characterise the sample, examine participant flow through the study, assess attrition rates at each stage, and evaluate satisfaction with the intervention. Descriptive statistics were also calculated for the primary and secondary outcomes, with data presented separately for each participant.

Reflexive thematic analysis (Braun and Clarke 2006, 2021c) was used to analyse the qualitative data due to its theoretically flexible approach. Reflexive thematic analysis values researchers' subjectivity in the analytic process and encourages researchers to actively consider how their assumptions and biases may shape interactions with participants and interpretations of the data (Braun and Clarke 2021a). ED conducted this study as part of her doctoral research that aims to increase access to psychological treatment for children and young people with eating disorders. The wider research team have extensive experience of delivering psychological treatments to children and young people with eating disorders and other mental health difficulties. The dataset was analysed inductively (directed by the content of the data) and semantically (reflecting the explicit content of the data).

Two researchers (ED and AD) independently familiarised themselves with the data and conducted line‐by‐line coding. Each transcript was coded by both researchers to promote objectivity, reliability and accuracy of the coding process (Braun and Clarke 2021b, 2023). A Cohen's kappa statistic of 0.87 indicated substantial agreement between coders. Initial codes were then grouped into potential themes, which were reviewed within the team to ensure that the interpretations reflected the data. Themes were refined and finalised through iterative revisions. *NVivo* was used to support data analysis and organisation.

## 3 Results

### 3.1 Recruitment

**FIGURE 1:**
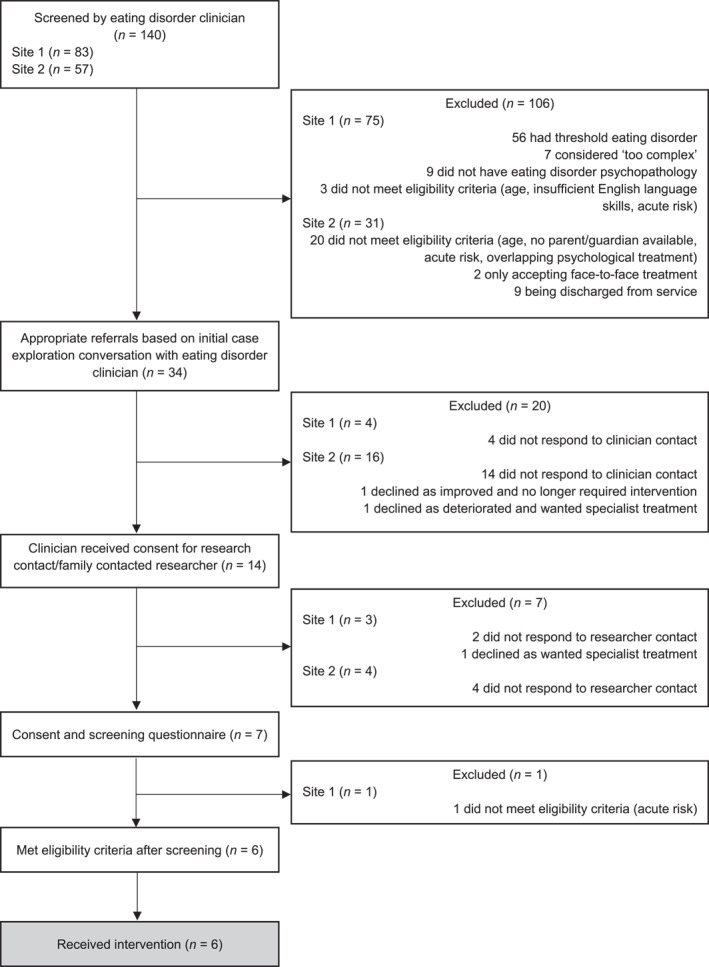
CONSORT flow diagram of participant recruitment.

During the 5‐month recruitment period from November 2023 to March 2024, 34 potentially eligible participants were identified across the two eating disorder services and contacted by a clinician at the respective sites. Of these, 14 families either permitted the clinical team to share their details with the research team or contacted the research team directly. Among the remaining 20 potential participants, 18 did not respond to clinician contact, and two declined to participate—one parent cited that their child had significantly improved and no longer required intervention, and the other stated that their child had deteriorated whilst on the waitlist for FBT and required specialist treatment. The research team subsequently contacted all 14 families who expressed an interest in the study. Six did not respond to this follow‐up contact, and one declined due to a preference for face‐to‐face treatment with a specialist. Seven families provided informed consent/assent to participate. However, one family was excluded during the screening stage due to acute risk. The final sample of participants consisted of six female adolescents and their primary caregivers, all recruited while on the waiting list for FBT at one of the sites (Site 2). A CONSORT flow diagram is provided in Figure 1.

### 3.2 Participants

Table 2 provides an overview of the demographic information for the six participants. The mean age of participants was 15.17 years (SD = 1.47; range = 13–17), and all were female and White British (100%). Five of the six participants came from nuclear families (83%). The index of multiple deprivation deciles of the sample, based on the English indices of deprivation, ranged from decile six (50%–60% least deprived) to decile nine (80%–90% least deprived). The participants presented with a range of DSM‐V eating disorder presentations: three had bulimia nervosa, two had anorexia nervosa, and one had OSFED (atypical anorexia nervosa). The duration of their illness ranged from 15 months to 3 years. At baseline, participants' %mBMI ranged from 78.80% to 95.50%. Five participants (83%) had comorbid anxiety and/or low mood, and two participants had Attention‐Deficit/Hyperactivity Disorder (ADHD).

**TABLE 2:** Demographic information of each participant.

	Age	Gender	Ethnicity	ED diagnosis	ED duration	%mBMI	Comorbid difficulties
A	13	Female	White British	OSFED	3 years	94.91%	ADHD
B	16	Female	White British	AN	16 months	90.51%	Social anxiety; generalised anxiety; low mood
C	16	Female	White British	BN	2 years	89.58%	Low mood; generalised anxiety
D	17	Female	White British	BN	15 months	95.50%	ADHD; low mood
E	15	Female	White British	AN	18 months	78.80%	Low mood
F	14	Female	White British	BN	15 months	80.49%	Generalised anxiety

Abbreviations: ADHD = Attention‐Deficit/Hyperactivity Disorder; AN = Anorexia Nervosa; BN = Bulimia Nervosa; ED = Eating Disorder; OSFED = Other Specified Feeding or Eating Disorder.

### 3.3 Feasibility and Acceptability

The average number of support sessions attended by participants was 7.67, ranging from six to nine sessions. Three parents were actively involved in the sessions. Each session lasted 33 min on average (range: 18–46 min). Overall, participants had an average total contact time of 4 h and 7 min with their guide, ranging between 3 h and 13 min (Participant D) to 4 h and 57 min (Participant A). Two participants were withdrawn from treatment early. Participant D sought counselling for low mood and was discharged from the study after completing six out of eight modules (excluding Modules 6 and 8). Participant E concluded treatment one session ahead of schedule (excluding Module 8) upon reaching the top of the waiting list for FBT at the eating disorder service. All young people and parents (100%) completed the baseline and post‐intervention assessments.

The results of the seven‐item acceptability questionnaire (Creswell et al. 2010) demonstrated that the treatment was acceptable to young people in this sample. Five young people (83%) either ‘agreed’ or ‘strongly agreed’ that they were satisfied with the programme and the support received, and four young people (67%) indicated that they would recommend (‘agreed’ or ‘strongly agreed’) the treatment to other young people. They particularly liked the patient stories within the modules, which helped normalise their difficulties. However, they almost unanimously reported that their least favourite aspect was the time required to complete each module and the practice tasks every week.

Similarly, parents in this sample also found the treatment to be acceptable, with five (83%) expressing overall satisfaction (‘agreed’ or ‘strongly agreed’). All participants (100%) responded positively (‘agreed’ or ‘strongly agreed’) regarding their satisfaction with the support received, and four parents indicated that they would recommend (‘agreed’ or ‘strongly agreed’) the programme to other families. Parents particularly valued the support sessions, as these reinforced their child's learning and helped to personalise the intervention. However, like the young people, parents critiqued the length of the modules and felt that one week was sometimes insufficient to work through the content.

There were no reported adverse events related to the intervention during the study.

### 3.4 Session‐By‐Session Measures

Session‐by‐session outcomes for each participant can be found in Supporting Information S2. Participants displayed a general upward trend in their GBO scores across the intervention period, indicating progress towards their goals (Figure S1). Participants displayed varied patterns in their ED‐15‐Y total scores throughout the intervention period (Figure S2). However, most (83%) showed a reduction in ED‐15‐Y scores between the first and final session. The pattern of SDQ SxS scores was inconsistent across the intervention period, although total impact scores were generally low for the cohort (Figure S3).

### 3.5 Baseline and Post‐Intervention Measures

Table 3 presents the self‐reported baseline and post‐intervention scores on the EDE‐Q, CIA, %mBMI, RCADS, and SDQ for all six participants. Parent‐reported outcomes of the scores of the PEDE‐Q, RCADS and SDQ are detailed in Table 4.

**TABLE 3:** Self‐reported scores on primary and secondary outcomes at baseline and post‐intervention for all participants.

	EDE‐Q^a^		CIA^b^		%mBMI		RCADS‐C^c^		SDQ^d^	
	Pre	Post	Pre	Post	Pre	Post	Pre	Post	Pre	Post
A	3.96	3.33	33	21	94.91%	100.19%	57	46	20	19
B	2.37	0.26	19	1	90.51%	98.60%	72	57	19	15
C	4.72	1.44	35	8	89.58%	98.73%	70	42	14	14
D	4.43	3.23	31	28	95.50%	95.50%	48	58	14	22
E	3.46	3.43	22	29	78.80%	76.90%	39	52	3	8
F	4.01	3.27	25	22	80.49%	87.46%	43	44	9	8
Mean	3.83	2.49	27.50	18.17	88.30%	92.90%	54.83	49.83	13.17	14.33
*SD*	*0.83*	*1.33*	*6.44*	*11.27*	*7.12%*	*9.08%*	*13.91*	*6.82*	*6.37*	*5.68*

^a^ EDE‐Q Global Score.

^b^ CIA Global Score.

^c^ RCADS Total Anxiety and Depression *t*‐score.

^d^ SDQ Total Difficulties Score.

**TABLE 4:** Parent‐reported scores on primary and secondary outcomes at baseline and post‐intervention for all participants.

	PEDE‐Q^a^		RCADS‐P^b^		SDQ^c^	
	Baseline	Post‐intervention	Baseline	Post‐intervention	Baseline	Post‐intervention
A	3.66	2.55	68	55	15	16
B	2.65	0.68	> 80	56	18	8
C	4.81	3.17	> 80	67	9	9
D	3.54	0.52	70	56	9	6
E	3.71	3.99	64	61	4	6
F	4.59	3.09	> 80	65	14	12
Mean	3.83	2.33	64.50	60.00	11.50	9.50
*SD*	*0.78*	*1.42*	*10.88*	*5.14*	*5.09*	*3.89*

^a^ PEDE‐Q Global Score.

^b^ RCADS‐P Total Anxiety and Depression t‐score.

^c^ SDQ Total Difficulties Score.

At baseline, the mean EDE‐Q global score was 3.83 (SD = 0.83, range = 2.37–4.72). At post‐intervention, the mean score decreased to 2.49 (SD = 1.33, range = 0.26–3.43). Similarly, parent‐reported EDE‐Q (PEDE‐Q) global scores showed a reduction from 3.83 (SD = 0.78, range = 2.65–4.81) at baseline to 2.33 (SD = 1.42, range = 0.52–3.99) at post‐intervention. Among the three participants who demonstrated binge eating, self‐induced vomiting and excessive exercise at baseline, there was a general reduction in the frequency of these behaviours at post‐intervention. There was also a reduction in the CIA global scores from baseline to post‐intervention for all participants except Participant E. The %mBMI increased for all participants from baseline to post‐intervention, again except for Participant E. The outcomes for the RCADS and SDQ were mixed; some participants scores improved (Participant A and B) while others worsened (Participant D and E). By the end of the study, Participants B, C and D felt that they had made considerable progress and no longer required additional support, so requested removal from the specialist eating disorder service waiting list.

### 3.6 Qualitative Feedback

Qualitative interviews were conducted with all young people (*n* = 6) and their parents (*n* = 6), yielding three overarching themes. Exemplar quotes relating to each theme can be found in Table 5. A more detailed overview of participant quotes for each theme is provided in Supporting Information S3.

**TABLE 5:** Representative examples of young person and parent qualitative feedback.

Theme	Feedback
Overall experience of treatment	“It was the best way to go about things when I wasn't getting help from anywhere else” (Participant B)
	“I think the initial benefits were that it’s something you can offer somebody immediately. Essentially, it's a really quick turnaround time rather than all the waiting times that there are currently” (Participant D's mother)
	“I think it's empowering for them to take charge of their own recovery in that way” (Participant F's mother)
	“The pie chart on all the things you have in your world right now, I found that quite a powerful exercise because it really shows that the majority of her life is focused on her appearance and weight. So it really showed me, and it showed her, that we need to put some more slices in the pie for her” (Participant E's mother)
	“I liked the part about [problem solving] and how effectively each strategy works. I didn't really think about it before, I just had like a bunch of strategies I do, but I didn't really think about how effective they are, and what might be the pros and cons of them” (Participant D)
	“I really like the sort of real personal contact, even though it's via teams. It kept [Participant A] motivated. It imposed deadlines” (Participant A's mother)
	“The practices that were taught in the module were helpful and the support sessions reminded me in my mind to follow through with the practices” (Participant C)
	“I attended all of the meetings with [Participant B] because that was helpful if [the guide] would make a suggestion. I Could put that into how we could bring that into our family life and how we could kind of make that happen for [Participant B] and work on those things” (Participant B's mother)
Impact of treatment	“Instead of dietary rules, I have like guidelines now” (Participant C)
	“She was in quite a dangerous place where she was restricting heavily and not seeing that she was deteriorating. She is in a much, much better place now and is able to take a much more balanced and informed approach, and is eating in a more healthy way now” (Participant C's mother)
	“Initially she was resistant and upset [about implementing the techniques], but yet, it has worked. It has reduced the amount of exercise she's doing. There's no silver bullet in 8 weeks. But I feel like she definitely has made some really positive steps she wouldn't have done before” (Participant A's mother)
	“The techniques for behaviours like binging, purging and that sort of thing led me to completely stop that. Learning about how it's just like a cycle and it's prone to keep on going and how you can work to stop that, I think that's really helped” (Participant F)
	“I think this has absolutely cemented some of the things that [Participant B] needed to do, and perhaps some of the things that she needed to take on board. And the big thing really is how giving her those strategies to cope when there's not… not every day is easy and some days you have tough days, but it's having those strategies to support her through some of those tough days” (Participant B's mother)
Suggested improvements to treatment	“Maybe a couple of sessions at the end where modules are repeated for things that they find particularly difficult” (Participant A's mother)
	“I think if it was a bit longer, the programme, it would be better because you're trying to embed in new ways of looking, thinking, feeling, behaviours and that's not very long to do it. What is it they say? It takes 12 weeks to create a new habit?” (Participant F's mother)
	“I feel like just making things shorter and more like simple. I mean, it is kind of already simplified, but less information written down rather than like loads of little questions about different things” (Participant D)
	“I think maybe videos would be helpful, and just making it more interactive and more exciting to look through. I Know that makes it sound like I'm like a little kid going through picture books, but just like making it more appealing” (Participant A)
	“I thought there wasn't enough on the emotions that you feel surrounding eating disorders” (Participant E)
	“I suppose because I did all the sessions with my mum, I think it could have been helpful if sometimes you did like the occasional session, just one on one, or just like 15 min one on one [with the guide]” (Participant E)
	“I really want to respect her confidentiality and her thoughts, but I think probably, I'd say for it to work, you need more parent involvement” (Participant D's mother)

Theme One, *Overall treatment experience*, encompassed participants' overall sentiments towards the treatment and treatment process. Participants valued the flexibility and convenience of remote delivery, as well as the ability to tailor the intervention through the selection and order of modules. Young people felt that the intervention helped them to make sense of their difficulties, encouraged them to achieve smaller goals, and challenged their maladaptive cognitions and behaviours. The self‐help approach empowered young people to implement changes, while the guide assisted them to personalise the strategies to their daily life. As half of the participants attended support sessions without their parents, it was difficult for some parents to comment on the intervention itself. However, parents who attended the support sessions found that the *“three‐way conversation” (Participant B's mother)* enhanced their ability to better support their child.

Theme Two, *Impact of treatment*, considered the intervention effects on the young person's eating disorder psychopathology and their general mental health and wellbeing. Participants reported that the intervention helped them to reduce dietary restraint, binge eating, and compensatory behaviours, as well as improve their body image. Additionally, they said that they felt more confident and that their communication skills had improved.

Theme Three, *Suggested improvements to treatment,* included recommendations from young people and parents to improve the intervention. Key suggestions included reducing the amount of text, incorporating more interactive elements like videos, and adding more content on emotions associated with changing eating behaviours. Additionally, they recommended optional support sessions for the young person without their parent, and for the parent without the young person, as well as providing refresher sessions to sustain progress.

## 4 Discussion

This proof‐of‐concept pilot study explored the feasibility, acceptability, and clinical impact of a transdiagnostic, CBT guided self‐help intervention for young people with eating disorders. Overall, the CBT guided self‐help intervention was feasible to deliver, generally well‐accepted, and associated with reductions in eating disorder psychopathology.

The intervention was successfully delivered to six young people on a treatment waitlist, all of whom completed at least six modules and six support sessions. Satisfaction ratings from young people and parents ranged from neutral to positive, consistent with findings from other eating disorder treatment studies for adolescents (DeBar et al. 2013; Hilbert et al. 2020; Manasse et al. 2021, 2024). Qualitative feedback highlighted the benefits of timely support, improved understanding of eating difficulties, and the acquisition of new techniques and coping strategies. The regular support sessions provided crucial guidance and structure, and enhanced accountability.

Notwithstanding the small, homogeneous sample, the preliminary results show promise for the CBT guided self‐help intervention in treating eating disorders in young people. At the group level, the intervention was associated with a reduction in eating disorder psychopathology, with EDE‐Q global scores decreasing from 3.83 to 2.49 (self‐reported) and 3.83 to 2.33 (parent‐reported). These results are largely consistent with outcomes observed in other brief interventions for adolescent anorexia nervosa (e.g., Lock et al. 2017; Thompson et al. 2023). However, given the modest sample size, these findings should be interpreted with caution. Since guided self‐help outcomes typically improve over time as individuals continue to implement strategies, we anticipate greater gains following the initial treatment period.

Intervention outcomes varied at the individual level. While most participants demonstrated improvements in eating disorder‐related outcomes, only half of the participants showed improvements in depression and anxiety symptoms. It may be unrealistic to expect that low intensity psychological interventions will be effective for all individuals with eating disorders. However, since robust predictors of outcome have yet to be established, it remains difficult to determine in advance who is most likely to benefit (McClure et al. 2024; Waller and Beard 2024). This intervention is not intended to replace individual therapy but to complement it within a broader care pathway (Davey et al. 2023). By freeing up skilled therapists to focus on patients who require more intensive interventions, it can enhance the efficiency and effectiveness of services. It has the potential to bridge gaps in service provision and provide timely support to young people who might otherwise face long waits for treatment or be unable to access care at all.

### 4.1 Strengths and Limitations

The current study has several notable strengths. Firstly, it is the first to pilot a transdiagnostic, CBT guided self‐help intervention for children and young people with eating disorders. Although similar interventions have been evaluated previously, such as FBT guided self‐help for adolescents with anorexia nervosa (Lock et al. 2021) and CBT guided self‐help for adolescents with bulimia nervosa (Schmidt et al. 2007), this study is, to our knowledge, the first to investigate a transdiagnostic CBT guided self‐help intervention for young people with eating disorders. Transdiagnostic interventions can address the heterogeneity and comorbidity that is often seen in real‐world settings (Levinson et al. 2022; Schaeuffele et al. 2021). The minimal exclusion criteria allowed young people with a range of eating disorder presentations to participate. However, it should be noted that binge eating disorder was not represented, and the sample did not include any males. Another key strength of the study is its ecological validity. The findings suggest that young people with eating disorders can be supported effectively while on the waiting list for specialist treatment (i.e., FBT), and can be transitioned to higher intensity treatment, when necessary, as illustrated by Participant E's case.

The findings of this study, while novel, should be considered in the context of several limitations. First, only descriptive statistics are reported due to the small sample size. Second, the lack of a control condition prevents definitive conclusions about intervention efficacy. Any symptom improvements cannot unequivocally be attributed to the CBT guided self‐help intervention, as effects could potentially be due to spontaneous remission. A more rigorous approach would have involved using a non‐concurrent multiple baseline single case experimental design (SCED; Watson and Workman 1981). This study design enables researchers to make causal inferences by investigating whether symptom changes occur consistently with the sequential introduction of an intervention across different participants. However, the waiting period for treatment raises ethical concerns. Third, the lack of a follow‐up period limits our understanding of the long‐term outcomes of the intervention. Fourth, the study included only six White female participants, aged 13–17, who were seeking treatment at the same eating disorder service in England. This small, homogeneous sample limits the generalisability of the study findings. The reliance on clinical teams to screen and refer participants, while ethically important, may have limited our ability to reach the target population consistently.

Additionally, the reliance on self‐reported outcome measures introduces potential biases, such as denial of symptoms or symptom severity (Vandereycken and Van Humbeeck 2008). Similarly, assessing weight online poses a limitation, as young people may not accurately report their weight. To enhance accuracy, parents were instructed to assist their child with weight measurement when possible. Parent‐reported outcome measures were included to gather different perspectives and help mitigate some of these biases. However, these measures are also prone to biases, as parents may over‐report or be less attuned to their child's symptoms and distress (Drury et al. 2023). Furthermore, the CIA (Bohn et al. 2008) has only been validated for use in adults and was modified slightly for the school‐aged sample in this study. It is also important to note that only three parents were actively involved in their child's support sessions, which may have limited their perspective on certain aspects of the treatment process.

### 4.2 Implications

Although preliminary, these findings suggest that an eight‐module CBT guided self‐help intervention, with less than 5 hours of contact time with a paraprofessional, can produce clinical benefits for children and young people with eating disorders. This approach is less‐resource intensive, which can offer advantages at the individual level and for the broader healthcare system. For the wider system, the ability to deliver the intervention via a paraprofessional—who is cheaper to employ and requires minimal training—extends beyond the dominant model of in‐person, individual psychological therapy at a clinic (Kazdin 2023). This has the potential to alleviate pressure on specialist services and bridge the demand‐capacity gap (Kazdin, Fitzsimmons‐Craft, and Wilfley 2017), and ultimately increase access to psychological support for children and young people with eating disorders. Timely access to treatment is crucial for good clinical outcomes (Flynn et al. 2021; Wonderlich et al. 2020).

Clinicians at both sites were permitted to use their clinical judgement alongside the eligibility criteria to determine suitability for the intervention. At Site 1, this judgement was applied broadly to refer only subthreshold cases, while at Site 2, the intervention was used as a waitlist option for routine cases. No participants from Site 1 may reflect a clinical view that guided self‐help is not suitable for patients with significant psychopathology who meet diagnostic criteria for an eating disorder. It is important to note that Site 1 were meeting access and waiting time standards during recruitment (i.e., only a 4‐week wait for face‐to‐face evidence‐based treatment). This variability resulted in differences in referral types between sites and has implications for the feasibility of integrating guided self‐help into routine clinical care where patients typically meet diagnostic thresholds. Future research should therefore investigate clinicians' perspectives on the suitability of CBT guided self‐help for young people with eating disorders and prioritise the consistent application of eligibility criteria across settings.

The qualitative interviews yielded several recommendations to improve the intervention. Key suggestions included reducing the amount of text, incorporating more interactive elements such as videos, adding more content on managing negative emotions associated with behavioural change, and offering refresher support sessions to sustain progress. Once the intervention has been refined, more rigorous pilot testing with a randomised design and larger, representative sample is warranted to further determine the feasibility, acceptability and preliminary effectiveness of the intervention (Skivington et al. 2021). Future research should aim to establish a priori criteria for feasibility and acceptability to strengthen the interpretability of these evaluations. Future research should also investigate the longer‐term effects of the intervention, for example, by offering it to young people on a waitlist exceeding six months and conducting follow‐up assessments at three and six months.

## 5 Conclusion

The results of this pilot study with six participants suggest that the CBT guided self‐help intervention may be feasible, acceptable, and associated with a reduction in eating disorder psychopathology for some young people with eating disorders. Given the small sample size and lack of control group, these findings must be interpreted cautiously. Nevertheless, these preliminary data are promising and suggest that this intervention could be a valuable strategy for increasing access to psychological treatment for children and young people with eating disorders. The next step should be a randomised pilot study with a larger, more representative sample and longer‐term follow‐up.

## Consent

Informed consent was obtained from all individual participants included in the study.

## Conflicts of Interest

The authors declare no conflicts of interest.

## Supporting information

Supporting Information S1

Supporting Information S2

Supporting Information S3

## Data Availability

Research data are not shared.
