# Supplementary material for: Guided Self‐Help Treatment for Children and Young People With Eating Disorders: A Proof‐Of‐Concept Pilot Study
Source: Eur Eat Disord Rev. 2025 Jan 2;33(3):595–607. doi: 10.1002/erv.3171 (PMC11965551; doi:10.1002/erv.3171)
Supplement: Supplementary file 1 — Supporting Information S1 [file ERV-33-595-s003.docx]

**Example Topic Guide for Young People and Parents**

*Please note that the questions below are examples of the types of questions that children, young people and parents/carers might be asked post-intervention (with the wording of the questions being modified accordingly). The interview will be semi-structured and by nature, flexible in response to individual needs.*

Aim: To explore young people, and parents/carers, experiences of the guided self-help intervention, with a particular focus on acceptability

1. **Introduction**

Establish the purpose of the interview and the limits of confidentiality

1. **Contextual information**

Review relevant contextual information with young person or parent/carer. For example:

- Age of child
- Diagnoses
- Number of support sessions received
- Who attended the support sessions

1. **Initial views of the treatment approach (e.g., when they first heard about the study, read the participant information sheet and/or spoke to the study researcher)**

- What were your initial views of this treatment?
- What were your initial thoughts on the possible benefits/challenges of this approach?
- Was there anything that you found particularly helpful/unhelpful at this stage?
- Was there anything else you would have liked at this stage?

1. **Experiences of receiving the treatment**

- Did your views of the treatment approach change once you started treatment?
- What did you like/dislike about the treatment approach?
- Probe for benefits for the young person/parent/family
- Probe for challenges for the young person/parent/family
- How did you/your child find implementing the treatment techniques at home?

1. **Relevance of treatment to family**

- Do you feel that the treatment has had any positive/negative impact on the difficulties it was aimed at helping with?
- Explore what the particular difficulties being targeted
- Probe what has improved for the young person
- Probe what has not improved for the young person
- Do you think the issues covered in the treatment were relevant to you/your child/your family?
- Probe what issues were relevant and why
- Probe what issues were not relevant and why

1. **Experiences of the mode, content and structure of the treatment (workbook and sessions)**

- How did you/your child find the treatment workbook?
- Probe for views on content, style, length
- Probe for any suggestions of things they would have found helpful/preferred to receive
- How did you/your child find completing the module activities?
- Explore understanding of between session tasks
- Probe for views on content, style, length
- Probe for any suggestions of things they would have found helpful/preferred to receive
- How did you/your child find the support sessions?
- Probe for views on content, frequency, duration, rapport
- Probe for whether they felt sufficiently supported to implement the strategies from the workbook
- Probe for any suggestions of things they would have found helpful/preferred to receive

1. **Broader acceptability of the approach**

- Would you recommend this approach to other families?
- Probe for reasons why/why not
- Were there any issues that were not covered in the treatment that you wish were covered?
- Probe what would have been helpful to cover
- Probe what impact they think covering these issues would have had
- What would you change about this approach?
- What would you have liked more/less of with this approach?
- Do you have any suggestions for improvement to the treatment?
- How have you found continuing to implement the techniques now the sessions have ended?

1. **Summarise the interview**

Probe whether there is anything else that the young person or parent wishes to add or comment on. Thank the person for their time.
