# Supplementary material for: Guided Self‐Help Treatment for Children and Young People With Eating Disorders: A Proof‐Of‐Concept Pilot Study
Source: Eur Eat Disord Rev. 2025 Jan 2;33(3):595–607. doi: 10.1002/erv.3171 (PMC11965551; doi:10.1002/erv.3171)
Supplement: Supplementary file 2 — Supporting Information S2 [file ERV-33-595-s002.docx]

| **Participant quotes for each theme and sub-theme** | | |
| --- | --- | --- |
| **Overall experience of treatment** | | |
| **Guided self-help as an approach** | *Increase access to support* | “It was big, you know, considering that we’ve been on a waiting for I don’t know how long, but the actual fact we were getting something. It was really heartening when we first found out about the study, you know, that something was actually going to happen” (Participant A’s mother)  “It was the best way to go about things when I wasn’t getting help from anywhere else” (Participant B)  “When [the study team] contacted me, I felt it was something that would be really beneficial because we weren’t getting anywhere on waiting list to get any support through the [eating disorder service] for [Participant B’s] condition” (Participant B’s mother)  “I thought [the intervention] would be good because it gives us some control over what was happening. I thought it would be good because we were having to wait a long time for the CAMHS intervention to start and she needed help right away” (Participant C’s mother)  “I think the initial benefits were that it’s something you can offer somebody immediately. Essentially, it’s a really quick turnaround time rather than all the waiting times that there are currently” (Participant D’s mother)  “We were at the point where we were just so grateful for any help” (Participant E’s mother) |
|  | *Self-efficacy* | “I think sometimes, and especially at 16, I think you kind of expect a magic wand to kind of make everything better. When we had the sessions with [the guide], it was very much about ‘you own this’ and ‘what you put in, you get out’. As much as I’m here to support, [the guide] was there to support and give guidance, [Participant B] had to own that and she had to own putting those strategies in place and understanding the modules and how they can support her in her recovery” (Participant B’s mother)  “I think [the treatment approach] is good because it gives her the information to think over herself, because if she’s told ‘you need to do this’, it’s not helpful. But if she’s given the information, it helps her to make up her own mind about it” (Participant C’s mother)  “I think it’s empowered [Participant C]. Because she was trying to sort it out before on her own, and she was trying to deal with it and she was trying to manage and it just wasn’t working. But with [the intervention], it’s given her some skills and empowered her, and that is very, very good. So I would definitely recommend it to other young people” (Participant C’s mother)  “I think it’s empowering for them to take charge of their own recovery in that way” (Participant F’s mother) |
|  | *Guided self-help as an approach* | “I also really enjoyed doing the modules and then having the time with [the guide] and then going it through it together” (Participant A)  “I actually really enjoyed [the support sessions] and I think it was good I did go through those because like it was nice to do the module and then talk about it, instead of it just being this online thing where you just do the modules and you don’t have any like meetings, like thinking about it afterwards. And it was really nice to talk to [the guide] just about my problems. So I think [the support sessions] were pretty good” (Participant A)  “I think right at the beginning I thought it was potentially a good approach for [Participant F] because [Participant F’s] very good at self-directed learning. So even from a really young age she would go online to research things she was interested in, and she’s quite independent really. So I thought this approach could work for her. I wasn’t sure about the CBT element of it, but actually I’ve come to see that’s worked really well for her” (Participant F’s mother)  “I think it was really good to have a different theme each week with little exercises and things to focus on, breaking it up like that and then checking in with [the guide], I think that worked really well” (Participant F’s mother) |
| **Intervention format** | *Remote delivery* | “Well before I’ve done this, I’ve had therapy and I’ve had life coaching and that’s all been quite full on and in person with another person, so it’s scarier than doing it online. I think it is quite helpful to do it online and also it doesn’t take up as much time to do it online as it is in person” (Participant A)  “It was actually quite nice to do it at home because it was easier and it felt more relaxed instead of having to be face to face with that person” (Participant A)  “I think it’s definitely good as well for people who are just starting to try to recover because I think this would be a lot easier than like actually going to see somebody” (Participant A)  “[The guidance sessions] were in the times that were convenient to us as a family” (Participant B’s mother)  “If [Participant B] had got to the top of the waiting list and we were going to have this therapy in the [eating disorder service] where we would have to travel 40 minutes there, 40 minutes back, and the time out of her day or out of our day. I’m working, [Participant B] is in the mix of GCSEs, I don’t think we would have been able to do that at this time because couldn’t be taking time out of school etc. to go to therapy when she’s about to sit GCSEs, whereas this was so much more convenient” (Participant B’s mother)  “We did [the guidance sessions] at the end of like my working day and also online. [Participant B] would come from school, so it didn’t interfere with schoolwork. It’s half an hour. We don’t have to travel anywhere. So online, that suited us perfectly” (Participant B’s mother)  “I liked how I could just do the worksheets and stuff in my own time and stuff, because I had like a week to do it whenever I liked. It suited me” (Participant C)  “The whole everything being online kind of thing that makes it a lot less personal” (Participant D)  “I think it’s quite nice to do it at home because you don’t necessarily have to even tell anyone you’re doing the practices, you can just do it when you want. It’s like your own safe space” (Participant C)  “Like the Zoom calls, they’re fine but it can also be more difficult. I think being in person can sometimes be quite helpful” (Participant E)  “I do think sometimes going to a [clinic] is good, doing it in person, but then I realise it’s not always feasible” (Participant E’s mother)  “I like the fact that it was online. I like the fact it was virtual. I think for teenagers, they’re so used to that, it really speaks to them. It makes it less intimidating as well. I think [Participant F] would find it really intimidating to be sitting in a room with her parents and her clinician talking about all of this stuff” (Participant F’s mother) |
|  | *Treatment modules* | “The way that it was structured was really helpful because it gives you like a briefing at the start of what it’s about and, you know, does this kind of apply to me. And then it goes into obviously giving you facts and information about what it’s talking about. And then it just gives you time to kind of read it and then gives you like a reflection at the end. I think it was all pretty clear because like the activities that were actually in the module, they were relevant to what it said just before. And if you don’t quite understand what they’re saying, you can always go back and read through it again and it becomes clear to you” (Participant B)  “In terms of layout, how it was pitched, I thought it was very user friendly” (Participant E’s mother)  “One of the [modules] that she did first with how the brain works, to do with food and eating and eating regularly, and how actually binge eating and restricting does affect you, those sort of things were really helpful and that was a good initial one to do for us because getting that straight, and then working on the deeper causes of all of this like self-image and those types of things was really helpful” (Participant C’s mother) |
|  | *Guidance sessions* | “With the length of the sessions, I thought it was the perfect time. I struggle to concentrate for like a long amount of time, so having just that half an hour session, you didn’t feel rushed, but it was a nice time to just have a nice chat about how things are going” (Participant B)  “With how frequent they were, I think it was great to do it like that same time every week because personally, I need that set time so I know what’s going on because that’s how I deal with work is having that set date and then the same time. It’s all very structured and we were allowed to do that and it was worked so well because it gave us enough time to put things into practice from the module or any task [the guide] gave us and then report back on it the next week and see what maybe we could do differently or what works really well for us” (Participant B)  “The sessions were run really well in terms of the time spent going over the last module, what you took from that, and then the preparation for the next module” (Participant B’s mother)  “I think [the guide] was very intuitive about the length and not making them too long and just keeping it sort of quite light and actually just accepting the way [Participant C] approached it and not pushing her and stuff. So I think they were fine and [Participant C] was quite happy to do those” (Participant C’s mother) |
|  | *Tailored approach* | “I know there was bits around inducing vomiting or using laxatives and stuff like that, but [Participant B] was able to say ‘those bits aren’t relevant to me’ and so she would be able to kind of skip over them. So I don’t think there was anything that we would say that the content wasn’t sort of aimed right or wasn’t relevant. Instead, we were able to say ‘that’s not something you need to properly concentrate on” (Participant B’s mother)  “I think the topics were really spot on. The only ones that weren’t was the overexercising or using laxatives or anything like that, and we just skipped that module so that was fine” (Participant E’s mother) |
|  | *CBT approach* | “Understanding how your behaviours can like affect your eating disorder and possibly make it worse, and breaking out of those bad habits, all of those were like super helpful because I struggle with those the most” (Participant B)  “[The CBT cycle] was really good because it showed how all your emotions cause those habits and then it zooms into what’s keeping [the eating disorder] going” (Participant E)  “When we met with [the eating disorder service], they were must into ‘you’ve just got to make your child eat’, ‘take control away from them’ and I didn’t really like that. I found that we were under so much stress at that point, and I just thought I can’t do that and I don’t think it’s going to help [Participant F]. So for me, this approach was obviously very different, it was a lot broader, you know, looking at themes and looking at different aspects, looking from different angles at the whole problem. So I was really welcoming of that. I thought it opens it up and doesn’t make it about just ‘you’ve got to eat, you’ve got to eat’ – that just feels like too intensive somehow. So I liked the way that it was just kind of more expansive” (Participant F’s mother) |
| **Intervention content** | *CBT techniques* | “It asked me questions that I just never thought about like not having. I like the one where it’s like I don’t have to think positive, but I also don’t have to think negatively about it. I can just be like in between, and I never really thought about that” (Participant A)  “I thought the black and white thinking [practice] was really helpful to have an understanding of what’s going on for her” (Participant E’s mother)  “I think [Participant E] found the [behavioural experiments] particularly helpful, where you picked an avoided food and then you scored yourself on what your fear was for it and then having it and how you felt, and all of that reflecting. I thought that was a good exercise to do. The only thing is because there are so many foods at the minute, it’s a lot, it’ll take a long time to sort of get through those foods” (Participant E’s mother)  ‘I like the worksheets, especially the experiment ones where I had to go out and do something because it proves a point to me that I didn’t think was true, which was like a realisation for me” (Participant F)  “I liked the part about [problem solving] and how effectively each strategy works. I didn’t really think about it, I just had like a bunch of strategies I do, but I didn’t really think about how effective they are, and what might be the pros and cons of them” (Participant D) |
|  | *Regular eating* | “There was a part of one module which is about how often you should eat, like you shouldn’t wait more than four hours before again or something like that. It really helped me because it kind of reassured me that I should be eating and it’s good for my body” (Participant C) |
|  | *Guidelines not rules* | “The things I take away from it was about remembering that these are not rules, these are guidelines, things can be adapted, and you know, flexible” (Participant E’s mother) |
|  | *Self-esteem pie chart* | “The pie chart on all the things you have in your world right now, I found that quite a powerful exercise because it really shows that the majority of her life is focused on her appearance and weight. So it really showed me, and it showed her, that we need to put some more slices in the pie for her” (Participant E’s mother) |
|  | *Patient stories* | “When it did those little stories on other people, I really did like that, I thought that was interesting” (Participant A)  “She liked the little stories and stuff like that, the kind of graphics broke it up a bit. She seemed to quite like the model and the way it was set out” (Participant A’s mother)  “I think the way that the modules were done were super helpful because they showed you other people’s stories, where they were like going through the same issues as you. They just put the issues that you’re going through in such an understanding way, because when that doesn’t happen you feel a bit alienated in the fact you don’t think that many people know what you’re going through. But the modules did it in such an understanding way it made you feel like normal for going through what you were doing through” (Participant B)  “I liked in the workbook where they would have like a little story about how someone else related to [the practices]” (Participant C)  “I think having some real life examples of young, you know the caricatures is helpful” (Participant C’s mother) |
|  | *Relapse prevention* | “You know, we have the modules, I could go back and look at them, which is helpful. I do think that I’ll have to remind myself of these things because obviously I probably will start to go back a little bit. So I’ve definitely got to encourage myself more to look back at the modules and like keep going” (Participant A)  “I think the last module that we did was how we can continue the support that we’ve had and how we can continue using that, and having a module about that was so helpful because it’s literally just thinking about how after the [intervention] is over, we can continue using it and there were so many great suggestions. And being able to write down the activities that I think would help me” (Participant B)  “There’s prevention moving forward in terms of ‘Okay, if I start to see some of these signs and triggers, what could I do differently this time to make sure I don’t get pulled into going back to where I was’” (Participant B’s mother)  “So what I’m hoping is that the stuff she’s learned to help herself, that she’ll be able to remember that and remind herself of that when she is, you know, having a bit of a low patch” (Participant C’s mother) |
| **Role of guide** | *Clarification* | “I think there was one where it wanted me to do this like graph or pie chart thing, and that was a bit confusing because I didn’t really know how to do that, but I cleared that with [the guide]” (Participant A)  “[The guide] would go through the modules and ask if there was anything that I didn’t understand, and if there was, we would go through it so she could explain it again to see if I could understand it in a better way, because she knew that it was important that I got all the information that I could from the modules” (Participant B)  “I think some of the questions and stuff on the worksheets, I didn’t really understand some of them, but when I had the sessions with [the guide], she’d reword it for me in a way that I’d understand” (Participant C)  “If I was unsure about something, I could just bring it up and then she would explain it” (Participant D)  “[The guide] was really helpful, like if we didn’t understand anything in the module that we would have to leave, we would go over it in the session which was good” (Participant E) |
|  | *Accountability* | “I really like the sort of real personal contact, even though it’s via teams. It kept [Participant A] motivated. It imposed deadlines” (Participant A’s mother)  “I have to say that I think if she hadn’t done the weekly meetings with [the guide], I don’t think it would have gone it done at all. So at least that kind of made it manageable, like she knew she had to do it and send it off to [the guide]” (Participant D’s mother)  “I think for [Participant F] that contact with [the guide] was really, really crucial. If it had just been information landing every week and a workbook, that would have not have worked” (Participant F’s mother) |
|  | *Personalisation* | “It allowed for responses to be talked through and made more personal to her situation” (Participant A’s mother)  “[Participant A’s] issues were I suppose a little bit different in that we have very controlled eating, but still eating meals, and her main obsession was the number of steps and exercise and that type of thing, that was where her really issues lie, and I think it was able to be adapted to that” (Participant A’s mother)  “[The guide] would say in the meetings, before when she set up the next module, she was obviously very aware of the content and [Participant B’s] needs, so [the guide] would say ‘you know, you might not want to do this bit, or that bit might not be quite so relevant for you at the moment’. So, [the guide] was able to kind of guide [Participant B] through bits she should concentrate on which was really helpful” (Participant B’s mother)  “I know [the guide] moved [the modules] around to work through where [Participant C] was at as well, which was again really helpful. Rather than saying ‘no, it’s got to be in this order’. Because if she’d have backed out of the course at some point, at least she’s done the ones most relevant to her first. So that was good that [the guide] was really flexible” (Participant C’s mother) |
|  | *Reinforcing learning* | “We would just talk about how I felt about [the module]. I felt like that was very helpful because how it actually positively impacts me after reading it and the stuff that I took away from it, talking about that helps. Like what you’ve actually learned from that and if you are using that since reading it, I think that was definitely really helpful” (Participant B)  “Some of the practices that were taught in [the module] were helpful and [the sessions] reminded me in my mind to follow through with the practices” (Participant C) |
|  | *Helping to implement strategies* | “Obviously it gave you the activities during the workbook, but going over it with [the guide], she helped me understand how I can actually put it into my daily life” (Participant B)  “And then some of the examples that [the guide] gave for some of the experiments I could do was quite helpful” (Participant E)  “Sometimes I say ‘well what would [the guide] say now? which is quite helpful, because it’s not me trying to impart something, I can put it into this third person” (Participant F’s mother) |
| **Parental involvement** | *Parent can assist in implementing strategies* | “I attended all of the meetings with [Participant B] because that was helpful if [the guide] would make a suggestion. I could put that into how we could bring that into our family life and how we could kind of make that happen for [Participant B] and work on those things. So I think that for me was definitely beneficial that we both joined the call. That sort of three-way conversation was definitely helpful” (Participant B’s mother) |
| **Impact of treatment** | | |
| Impact of treatment on eating disorder symptoms | *Improved eating-related difficulties* | “[The intervention] was helpful and I’m glad I did do it because it did help and I also learned new things and I feel like it made me progress on to like getting better” (Participant A)  “Initially she was resistant and upset [about implementing the techniques], but yet it has worked. It has reduced the amount of exercise she’s doing. There’s no silver bullet in eight weeks. But I feel like she definitely has made some really positive steps she wouldn’t have done before” (Participant A’s mother)  “I would describe myself as like basically almost fully recovered. I obviously still have a couple of things that just make it like a little bit difficult” (Participant B)  “I can honestly say that I’ve seen a difference in [Participant B]. Although she was on that road to recovery, I’ve definitely seen a difference in her since we’ve done this [intervention]. I think that is down to what we’ve done over the last weight weeks” (Participant B’s mother)  “I think the [techniques] are just part of my normal routine now so I have managed to carry on doing them [since the intervention ended]” (Participant C)  “It’s given her some skills and put her in a position where she wanted things to change. But when she started on the course, she was in a pretty difficult position where she wasn’t thinking as clearly because of being very restricted. So I think the progress that she has made has been very good given the position she was in” (Participant C’s mother)  “I think a lot of the techniques that have stuck. There’s a lot of things, unhealthy things that I used to do that I now don’t” (Participant F)  “[Participant F] has a group of friends and they have a lot of sleepovers together, that was a big, stressful thing and there was a period of time when she was really unwell where she didn’t go because she was so worried they’d all bring loads of snacks, and it would all be kind of pizzas and things. And she was too worried about overeating, so she excluded herself. Or there was a period when she was going and then just losing control, and then getting quite freaked out by that. I think [the guide] made some really good suggestions about pace, ways to pace herself more and to reflect before she just went into one extreme or another. She seems less extreme than she was now, like by the starving or bingeing, and I think that’s been helped a lot by this programme” (Participant F’s mother) |
|  | *Reduced dietary restraint* | “It was worthwhile doing because it did mean that for one week she had to eat some bacon and stuff like that, which originally she was not happy doing. It helped around the peripheries of it, but not the sort of main issue with food and the way she restricts her diet” (Participant A’s mother)  “I genuinely can’t think when it really bothers me, like food as a whole, I can’t think when it really bothers me. Like I think sometimes like ‘oh, I’ve eaten like a lot today’. But then I catch myself like straight away and I go through what I have eaten and then I’m like, ‘actually I haven’t’. And that’s only like a minor part of what I’m thinking, because I just don’t think about it anymore. But when I do think about stuff like that, I’m able to break it down and realise I haven’t. And it’s just about like fully getting rid of those little bad habits. Overall, I know that I have definitely got a lot better than I was” (Participant B)  “Instead of dietary rules, I have like guidelines now” (Participant C)  “She has taken on board some of the ideas and thought about them and put them into practice, you know, like the eating regularly and how not eating affects your brain and stuff. We’d been told that but she hadn’t heard that information apart from in a book, so actually having a more accessible format means she can choose to listen to it or not to listen to it, but atleast its in a more accessible format” (Participant C’s mother)  “I think she’s got better with the eating side of it. She tries to eat regularly now because she doesn’t want to binge. I think that side of things have stuck” (Participant C’s mother)  “[Participant C] seems better and her eating is so much better. She is adapting to family meals, she is taking part in them. She’s dropped trying to eat all of the sort of low calorie foods and less sort of talking about that. She is eating more regularly, and she’s eating foods which before were too risky for her. So the foods that she would think ‘I’m going to binge on this so I won’t eat it at all’. She’s eating some of those foods but she’s eating them at a more, you know, sensible amount rather than the binge which is too much. So she’s eating with a normal range of volume a lot of the time. She’s still probably not quite eating as much as maybe she should, but every day she’s eating within a reasonable range. She’s so much better and, you know, she’s very proud of her progress” (Participant C’s mother)  “She was in quite a dangerous place where she was restricting heavily and not seeing that she was deteriorating. She is in a much, much better place now and is able to take a much more balanced and informed approach, and is eating in a more healthy way now” (Participant C’s mother)  “I would say she seems a lot less extreme and she talks about recovery more. I remember going shopping with her a couple of weeks ago and she saying ‘oh no, they’re not good to buy’, they were like gyozas or something… ‘they’re really low calorie, they’re not good for recovery’, which was a really big step you know, normally she’d be wanting that kind of food that was really low calorie” (Participant F’s mother) |
|  | *Reducing bingeing and purging* | “The techniques for behaviours like binging, purging and that sort of thing led me to completely stop that. Learning about how it’s just like a cycle and it’s prone to keep on going and how you can work to stop that, I think that’s really helped” (Participant F) |
|  | *Improved body image* | “She still likes her comfy days and baggy clothes, but if she’s going out and seeing her friends, she definitely pushes herself a little bit more to kind of say ‘right, actually, I’m gunna wear this, and I’m gunna feel comfortable in wearing this’. So yeah, I think there’s some really good strategies that supported us” (Participant B’s mother)  “[Participant C] is feeling more positive about her body. Yes, it’s not as far as it should go. She is able to go into busier places so she’s feeling less socially anxious than she was. But yeah, there’s still a way to go. She’s slightly more open to even having a picture taken now than she was” (Participant C’s mother)  “I think early on [the guide] kind of said to [Participant F] that she was kind of fragmenting herself, she wasn’t seeing herself as a whole person, she was fixating on when she was very anorexic. She was really fixating on different bits of her body and, you know, thinking that that part of her body was overweight or whatever. I think she does still do that to some extent but I think it’s less and she has more awareness around it” (Participant F’s mother) |
| Impact of treatment on general mental health and well-being | *Improved confidence* | “I just feel like reading through [the modules] and also doing the questions and stuff, it just helped me feel more confident and just better about myself” (Participant A)  “I think that’s probably the biggest thing, it’s had a big impact on her own self-confidence and her confidence in her image” (Participant B’s mother) |
|  | *Improved communication* | “The big thing for [Participant B] actually, was that she couldn't always understand and perhaps deal with challenges herself, and she would take a long time to come to me. I could tell that something is wrong, but it would take a long time for her to actually find the confidence or find the words to explain how she felt. Whereas now I can see something's wrong and I'll say what's the matter and she might not just come out with it straight away, but she will start to talk and then start to give much more detail. She is able to communicate better and express how she's feeling a little bit better. I mean that’s definitely been in the last sort of eight weeks really. Before that, it could have gone on for hours. I kept saying ‘what’s wrong, what’s wrong?, and then she just didn’t know how to put into words, whereas I think now she’s got a little bit more understanding of some of the things that she is experiencing, some of the things that she’s feeling, and then is able to communicate that little bit better” (Participant B’s mother)  “I was learning as well, I was finding out things and it was a good jumping off point for conversations which was really useful” (Participant E’s mother)  “I mean on the whole, it was positive really because we were able to have conversations about the things and all the topics evolved into conversations which were really useful” (Participant E’s mother) |
| **Suggested improvements to treatment** | | |
| **Critique of intervention** | *Duration of programme* | “I suppose like the time you get given, I think it was eight weeks, I suppose it does seem like kind of a short amount of time to go over all that stuff” (Participant E) |
|  | *Time-consuming* | “I did find them a tiny bit long because I normally did them after school and I’d just be scrolling through them being like ‘this is so much work to have to read through all of this and then complete the questionnaires’. But I mean it was helpful and I’m glad that I did do it. It was just a bit long really, but I understand it’s like a minor inconvenience” (Participant A)  “I think the problem was that in her mind it was a bit like homework, so it sort of got let a bit until the last minute and then sort of rattle through it as quickly as possible. I think that might just be a [Participant A] thing rather than anything else, you know? I don’t know how you get round that because it’s literally ‘this is your module, you have to do it by this date’ and then we have to submit the feedback. So I think it’s something she had to kind of get on board with” (Participant A’s mother)  “[Participant B] would do the module, some of them very long, and I think there’s a lot of reading within that and you know, I do know that [Participant B] probably skipped through some things and then went to some elements that were a bit easier on the eye or not so word heavy” (Participant B’s mother)  “[Participant C] commented that the [workbooks] did take a while to do and that sort of thing, that some of them were quite long. Some of the ones I saw were quite lengthy, and you’ve got these extra exercises that you’ve got to try and do which she would do. But obviously it’s quite a lot of time, and given the circumstances that she was in, it was just quite a bit” (Participant C’s mother)  “I felt like I had so much to do every week and a lot of it was kind of very similar, and while I was doing it I was just kind of thinking about other work I should be doing” (Participant D)  “Some of [the modules] were quite long and it’s a lot of information to do over the week” (Participant E)  “Sometimes the modules felt quite long and a lot to get through in the week, so that felt like a big pressure” (Participant E’s mother)  “With everything else that’s going on with an eating disorder, it’s picking your moments and trying to find time to do it, amongst you know the rest of the family as well. So some of the modules were quite big, it felt like quite a lot to get through, especially as we really wanted to take full advantage of it” (Participant E’s mother) |
|  | *Relevancy of modules* | “I felt like quite a bit of it was to do with binge eating and like throwing up, that’s not relevant to me, and I feel like maybe it could be more about exercising and less about laxatives and throwing up and binge eating, because that just wasn’t really relevant to me” (Participant A)  “Some [modules] were more relevant than others, but we understand they have to cover everybody. You know, it talked about vomiting and stuff like that, and that has never been her issue, nor is she taking laxatives or anything like that. So because of that, you know, there were certain things we had to kind of skip over” (Participant A’s mother)  “I think there’s obviously going to be some things that were mentioned that weren’t relevant to me, like bulimia and then binge eating, and stuff like using laxatives, and then also with weighing, that wasn’t so much of an issue with me. I’m not bulimic, I don’t binge eat, I don’t use laxatives”(Participant B)  “The reason I didn’t go through the [whole programme], I like left in the middle, was because I got like therapy. And I realised a lot of my problem with the whole eating is because like of other stuff on top of it. So like I think what worked better was talking about like whatever else was stressing me out as well as the eating sort of stuff, whereas this [intervention] was just about eating disorder so it wasn’t as relevant for me” (Participant D)  “I think some modules were more helpful than others because not all the modules I feel like are relatable to everyone because everyone’s experience is different” (Participant E)  “Some of the questions I felt like weren’t very beneficial to me, so those ones I kind of didn’t answer, and stuck with the ones that I thought I was gunna get the most out of” (Participant E)  “I suppose it’s like with anything, not absolutely everything is gunna fit” (Participant E’s mother) |
|  | *Modules were text-heavy* | “It isn’t gunna be the case for everyone, but I’m dyslexic, so sometimes reading all of the information, I found that like stressful when it would just confuse me” (Participant D) |
|  | *Difficult to find a private space for guidance sessions* | “It probably would have been better if I went on a walk and sat somewhere else because I didn’t wanna be overheard when I was talking. I felt like I could be intruded on and I’d be like stressed about like thinking about it” (Participant D)  “I think sometimes actually going to a [clinic] does help, you know? But also being around at home, obviously it has it’s benefits as well, but it’s trying to sort of navigate some private time way from the rest of the family. So that was quite hard sometimes to make sure we had some time set aside so wouldn’t be interrupting anything else” (Participant E’s mother) |
| Changes to modules | *More content on emotions* | “I thought there wasn’t enough on the emotions that you feel surrounding eating disorders” (Participant E) |
|  | *More interactive (e.g., videos)* | “I think maybe videos would be helpful, and just making it more interactive and more exciting to look through. I know that makes it sounds like I’m like a little kid going through picture books, but just like making it more appealing” (Participant A)  “I guess if I was gunna say anything, a few more videos or something, that could have gone in [the intervention], like bitesize videos that would kind of capture [the information]. She does enjoy reading, but I think some of them were like 15 pages long, you think… ‘keep going, keep going’” (Participant B’s mother)  “Sometimes I think a lot of kids find it hard to soak in pure information, so like videos and stuff like that I think would be more helpful, like scenarios” (Participant F) |
|  | *Shorter in length* | “Videos and just shorting the modules down a little bit. Other than that, I think it was really good” (Participant A)  “I think the only thing is some of the modules could be quite long and I think you know concentration wise, just perhaps some different sort of ways of going through stuff in terms of like videos or podcasts, or something like that might kind of break them up a little bit. But again, it didn’t have an impact on how [Participant B] found it or anything like that, it would just be something that perhaps would have made it a little bit more user friendly” (Participant B’s mother)  “So I think it depends how much available time you’ve got, but may be splitting it down to smaller amounts of some of the longer [modules] may have been better” (Participant C’s mother)  “I think the only thing is whether any of [the modules] could be broken down a little bit. It felt like quite a lot to get through, whereas if they were split, we have been able to focus in a bit more” (Participant E’s mother)  “Some of [the modules] are 18/19 pages, and whether they could be just broken down a little bit more” (Participant E’s mother)  “I think you don’t wanna overwhelm and overload you know, because they’re already in a state of overwhelm with all of this. So I wouldn’t say you’d want more [content] for sure, if anything, just space it out and make it longer and less. So more bite-sizey chunks, longer time to digest and integrate” (Participant F’s mother)  “I think to have that ongoing support but for a longer period. I don’t think you would necessarily need to add any more content in, it would just be more of a case of expanding the content to a wider timeframe” (Participant F’s mother) |
|  | *More personalisation* | “Some of the modules were a lot more information than some of the others which I suppose I think I could have done with more time working on those ones, or more time on the modules which I related to the most and the less time on the others” (Participant E)  “I suppose just making the modules more individual, but I know that’s quite hard to do. But I think just narrowing the modules down to each person so they can get the most out of it that they can” (Participant E) |
| Changes to guidance sessions | *Refresher sessions* | “I suppose if I was being greedy, I would say for it to last longer than 8 weeks maybe. So, for example, having a more positive body image. Yes, she has followed someone who is more positive on Instagram and things like that. But just sort of keeping on top of that, because I think it’s very easy to sit back and your feed to get the same as it was before. So maybe a couple of sessions at the ends where modules are repeated for things that they find particularly difficult” (Participant A’s mother)  “I would like her to have some follow up or something like that just to say like ‘how are things going? Is there any follow-up on anything you need?” (Participant C’s mother)  “I think my concern really is ‘what now?’. You know it’s quite quick to go through so I think if you know you could have spent a month on each topic or two weeks even or it nuanced it more for each topic, I think if it was a bit longer, the programme, it would be better because you’re trying to embed in new ways of looking, thinking, feeling, behaviours and that’s not very long to do it. you know, what do they say – it takes 12 weeks to create a new habit” (Participant F’s mother)  “I think she had an extra week on one of the topics because of various thing, but I think that was too short. But having said that, I still think it has definitely had an impact” (Participant F’s mother)  “[Participant F] in the last few days has had a dip. She’s quite down at the moment and I think because she is recovering more, she’s put a bit of weight on. We went away to Italy and we had loads of lovely food and things and she had a few dips where we were away. My concern really is more now it’s finished. And when I brought that up to [the guide], she said ‘she’s got the resources’ but it’s not the same. Just having information isn’t enough, I don’t think. And if you’re really trying to change and work with something that’s complex and difficult, it’s good to have the resources, but it’s not the same as being held in a process and checking in with somebody and having that support. So I think it would be worth considering, even if it was 12 weeks” (Participant F’s mother)  “I think a follow up is really needed. You know, what people do at the end of this kind of thing, that needs more thought. Just saying ‘you’ve got the resources’ isn’t enough” (Participant F’s mother)  “I just think the programme needs to be, it was really good, but I think it was too short and I think a way to support more would be to have the same amount of content, but spread it out longer” (Participant F’s mother)  “I think maybe where you would do the programme and then you would revisit it, because it’s a lot to take on and a lot to change. But then to have some kind of follow-up process, maybe down the line, three months, six months later or something, just to see if there is any residue that needs to be kind of dealt with, or you know, areas that haven’t really kind of worked or stuck or been able to shift. I think that would be very helpful if that was possible” (Participant F’s mother) |
|  | *Cover more content in session* | “I think for preference I would do all the stuff I need to do in the call, which I know isn’t how it’s supposed to work” (Participant D) |
|  | *Longer in duration* | “I think some sessions could do with being a little bit longer if there’s like more information to cover” (Participant E)  “We had like a half an hour so I think perhaps a longer session to catch up as the half an hour seemed to go in a blink of an eye, especially if there was a lot in that module. And sometimes it took [Participant E] a little while to warm up” (Participant E’s mother) |
| Involvement of parents | *More parental involvement* | “You know, we’ve gone on this journey as a family. When the intervention stops, we are her support network and it’s good if we’re informed about the right things as well, and that we’re not inadvertently being unhelpful as well. It is as much as educating us actually, because we could be reinforcing some of those negative thoughts” (Participant C’s mother)  “I would have like to have done it with her. I would have liked to have sat down with her being in the sessions, potentially had a little bit where she’d be able to say that she’ll ‘just answer this bit of my own mum’ and I would have been totally fine with that” (Participant D’s mother)  “I really want to respect her confidentiality and her thoughts, but I think probably, I’d say for it to work, you need more parent involvement” (Participant D’s mother)  “So there is obviously supporting the young person or the child, but also supporting the parents a bit more actually. And I think maybe [the guide] left it to me to be in touch with her, but I didn’t, it wasn’t kind of in my awareness enough I don’t think. I think if it had a bit more structure around it for myself or from her or from the programme, I would have reflected more and engaging in different ways which I think would have been helpful. But I did really feel that [Participant F] wanted it as her thing. So I kind of stepped right back, but I think it could have still been her thing and I can have had my thing a bit more” (Participant F’s mother)  “I think for the parents to get support because it’s really stressful and upsetting as a part to be in this, less so now, but it has been really stressful at times and to kind of get some of that support back as well and guidance, sometimes a small thing can have a big impact. You know, a little steer on something can actually be reassuring or just widen your lens a bit, it will calm part of you down that’s really worried, so I think that actually would be a huge benefit” (Participant F’s mother) |
|  | *1:1 session for young person without parent* | “I suppose because I did all the sessions with my mum, I think it could have been helpful if sometimes you did like the occasional session, just one on one, or just like 15 minutes one on one [with the guide]” (Participant E) |
|  | *1:1 session for parent without young person* | “I think in the beginning I discussed it with [Participant E] and she was quite keen for me to be there. But sometimes I can’t speak freely as I’m sort of filtering what I say, so that was the only thing. I know [the guide] was at the end of an email, so there was a little room there to chat to her without [Participant E]” (Participant E’s mother) |
