# Supplementary material for: Guided Self‐Help Treatment for Children and Young People With Eating Disorders: A Proof‐Of‐Concept Pilot Study
Source: Eur Eat Disord Rev. 2025 Jan 2;33(3):595–607. doi: 10.1002/erv.3171 (PMC11965551; doi:10.1002/erv.3171)
Supplement: Supplementary file 3 — Supporting Information S3 [file ERV-33-595-s001.pdf]

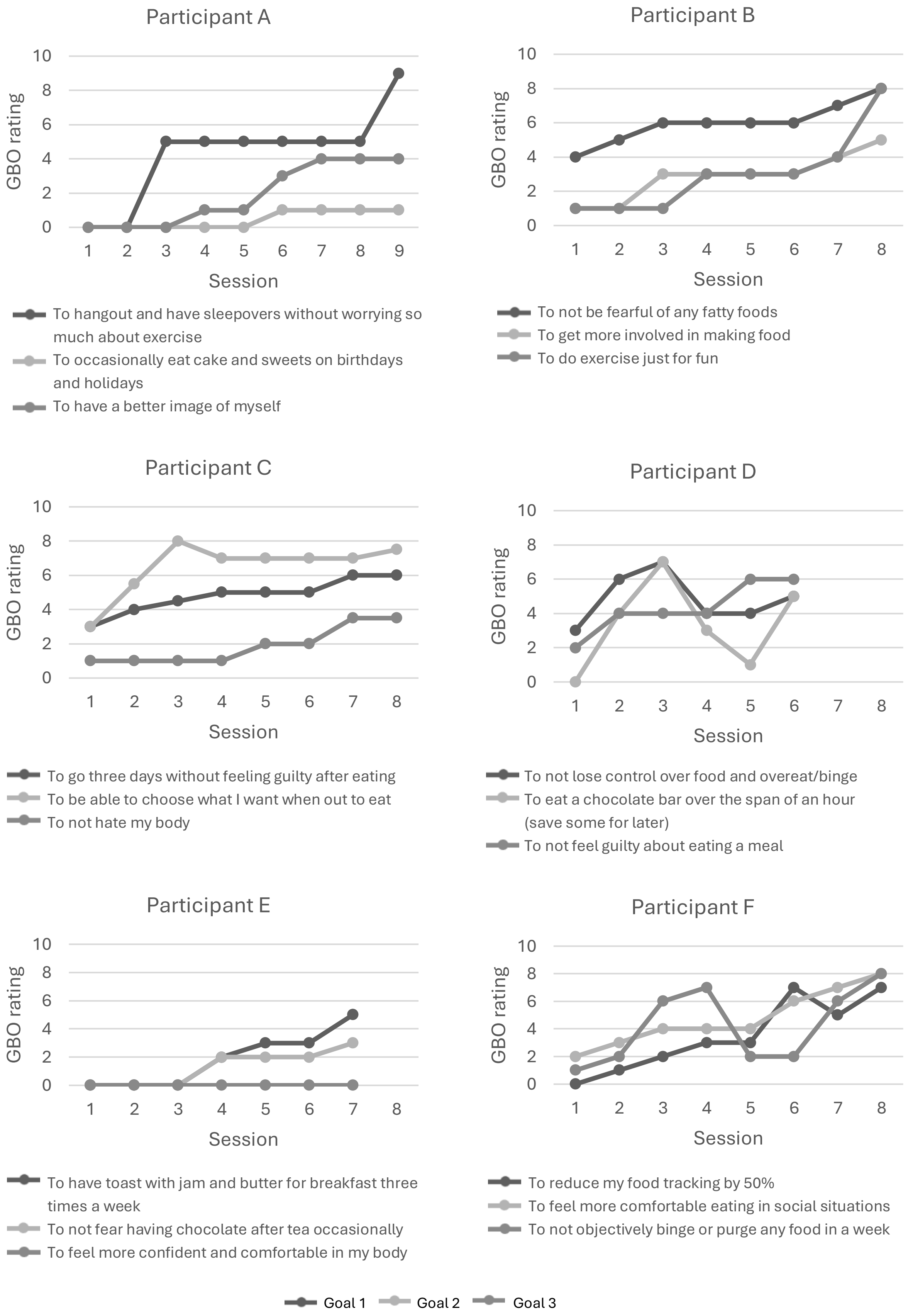

Figure S1. Session-by-session Goal Based Outcomes (GBOs) for each participant.

Note. Higher ratings (closer to 10) indicate greater progress towards goal.

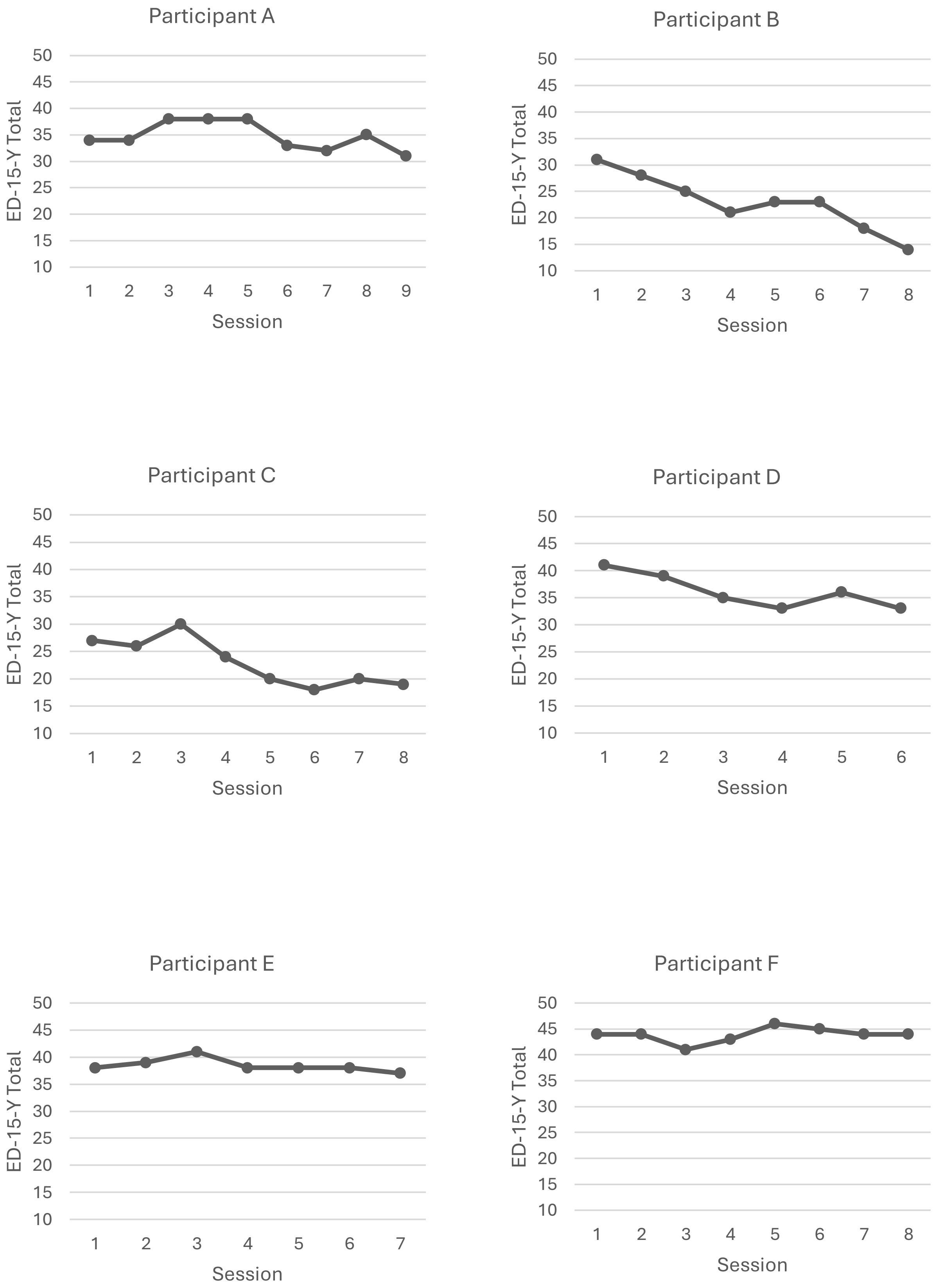

Figure S2. Session-by-session ED-15-Y total scores for each participant.

Note. Lower total scores (closer to 0) indicate better outcomes.

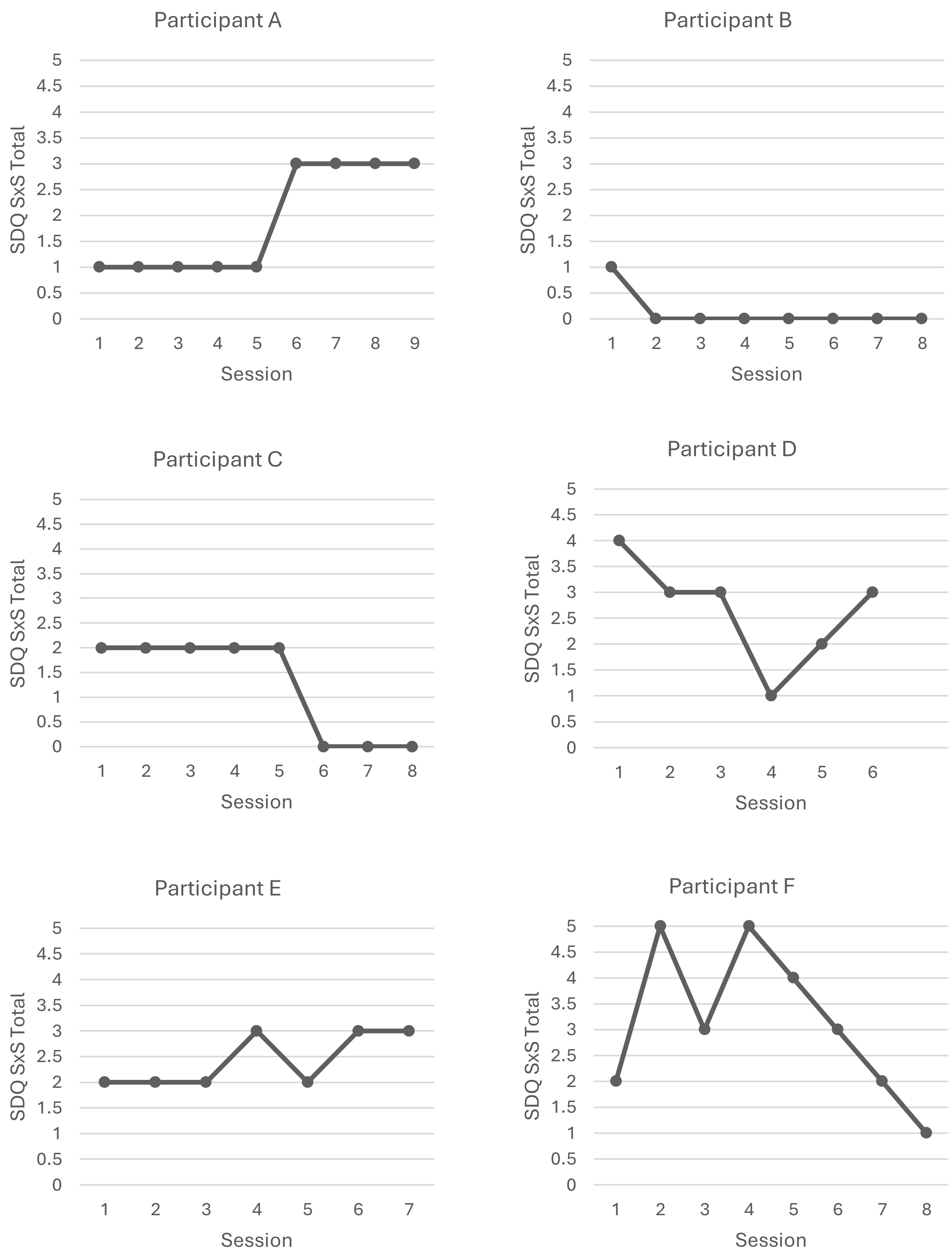

Figure S3. SDQ SxS total scores for each participant.

Note. Lower impact scores (closer to 0) indicate better outcomes.
